# FAIRsharing as a community approach to standards, repositories and policies

**DOI:** 10.1038/s41587-019-0080-8

**Published:** 2019-04-02

**Authors:** Susanna-Assunta Sansone, Peter McQuilton, Philippe Rocca-Serra, Alejandra Gonzalez-Beltran, Massimiliano Izzo, Allyson L. Lister, Milo Thurston, the FAIRsharing Community

**Affiliations:** Oxford e-Research Centre, Department of Engineering Science, University of Oxford, Oxford, UK

**Keywords:** Education, Research data, Research management, Data publication and archiving, Standards

**To the Editor** — Community-developed standards, such as those for the identification^1^, citation^2^ and reporting^3^ of data, underpin reproducible and reusable research, aid scholarly publishing, and drive both the discovery and the evolution of scientific practice. The number of these standardization efforts, driven by large organizations or at the grassroots level, has been on the rise since the early 2000s. Thousands of community-developed standards are available (across all disciplines), many of which have been created and/or implemented by several thousand data repositories. Nevertheless, their uptake by the research community has been slow and uneven mainly because investigators lack incentives to follow and adopt standards. Uptake is further compromised if standards are not promptly implemented by databases, repositories and other research tools, or endorsed by infrastructures. Furthermore, the fragmentation of community efforts results in the development of arbitrarily different, incompatible standards. In turn, this leads to standards becoming rapidly obsolete in fast-evolving research areas.

As with any other digital object, standards, databases and repositories are dynamic in nature, with a ‘life cycle’ that encompasses formulation, development and maintenance; their status in this cycle may vary depending on the level of activity of the developing group or community. There is an urgent need for a service that enhances the information available on the evolving constellation of heterogeneous standards, databases and repositories; guides users in the selection of these resources; and works with developers and maintainers of these resources to foster collaboration and promote harmonization. Such a service is vital to reduce the knowledge gap among those involved in producing, managing, serving, curating, preserving, publishing or regulating data. A diverse set of stakeholders, representing academia, industry, funding agencies, standards organizations, infrastructure providers and scholarly publishers—both national and domain-specific as well as global and general organizations—have come together as a community, representing the core adopters, advisory board members, and/or key collaborators of the FAIRsharing resource (https://fairsharing.org/communities). Here we introduce its mission and community network. We evaluate the standards landscape, focusing on those for reporting data and metadata and their implementation by databases and repositories. We report on the ongoing challenge to recommend resources and the importance of making standards invisible to the end users. Finally, we highlight the role each stakeholder group must play to maximize the visibility and adoption of standards, databases and repositories.

## Mapping the landscape and tracking evolution

Working with and for data producers and consumers, and taking advantage of our large network of international collaborators, we have iteratively^3–5^ developed FAIRsharing (https://fairsharing.org), an informative and educational resource that describes and interlinks community-driven standards, databases, repositories and data policies. As of February 2019, FAIRsharing has over 2,620 records: 1,293 standards, 1,209 databases and 118 data policies (of which 82 are from journals and publishers and 23 from funders), covering natural sciences (for example, biomedical, chemistry, astronomy, agriculture, earth sciences and life sciences), engineering, and humanities and social sciences.

Using community participation, the FAIRsharing team precisely curates information on standards employed for the identification, citation and reporting of data and metadata, via four standards subtypes. First, minimum reporting guidelines—also known as guiding principles or checklists—outline the necessary and sufficient information vital for contextualizing and understanding a digital object. Second, terminology artifacts or ‘semantics’, ranging from dictionaries to ontologies, provide definitions and unambiguous identification for concepts and objects. Third, models and formats define the structure and relationship of information for a conceptual model and include transmission formats to facilitate the exchange of data between different systems. And lastly, identifier schemata are formal systems for resources and other digital objects that allow their unique and unambiguous identification. FAIRsharing monitors the evolution of these standards, their implementation in databases and repositories, and recommendation by journal and funder data policies.

Producers of standards, databases and repositories are able to claim the records for the resources they maintain or have developed; this functionality allows them to gain personal recognition and ensures that the description is accurate and up-to-date. All records and related updates by the maintainers are checked by a FAIRsharing curator. Conversely, if a record is updated by a FAIRsharing curator, an e-mail notification is sent to the record claimant, minimizing the introduction of inaccuracies. In communication with the community behind each resource, FAIRsharing assigns indicators to show the status in the resource’s life cycle: ‘Ready’ for use, ‘In Development’, ‘Uncertain’ (when any attempt to reach out to the developing community has failed), and ‘Deprecated’ (when the community no longer mandates its use, together with an explanation where available).

To make standards, databases, repositories and data policies more discoverable and citable, we mint digital object identifiers (DOIs) for each record, which provides a persistent and unique identifier to enable referencing of these resources. In addition, the maintainers of each record can be linked with their Open Research and Contributor IDentifier (ORCID) profile (https://orcid.org). Citing a FAIRsharing record for a standard, database and repository offers an at-a-glance view of all descriptors and indicators pertaining to a resource, as well as any evidence of adoption or endorsement by a data policy or organization. Referencing the record together with the resource’s main paper (which provides a snapshot of its status at a given time) provides a complete reference for a resource. FAIRsharing has its own record to serve this very purpose: 10.25504/FAIRsharing.2abjs5.

FAIRsharing collects the necessary information to ensure that standards, databases, repositories and data policies align with the FAIR data principles^6^: Findable (for example, by providing persistent and unique identifiers, and functionalities to register, claim, maintain, interlink, search and discover them), Accessible (for example, identifying their level of openness and/or license type), Interoperable as much as possible (for example, highlighting which repositories implement the same standards to structure and exchange data) and Reusable (for example, knowing the coverage of a standard and its level of endorsement by a number of repositories should encourage its use or extension in neighboring domains, rather than reinvention). FAIRsharing collaborates with many other infrastructure resources to cross-link each record to other registries, as well as within major FAIR-driven global initiatives, research and infrastructure programs, many of which are generic and cross-disciplinary. A ‘live’, updated list is maintained at https://fairsharing.org/communities, with the roles that FAIRsharing plays. An example is the FAIR Metrics working group (http://fairmetrics.org)^7^, where we work to guide producers of standards, databases and repositories to assess the level of FAIRness of their resource. We will develop measurable indicators of maturity, which will be progressively implemented in the FAIRsharing registry.

The content within FAIRsharing is licensed via the Creative Commons Attribution ShareAlike 4.0 license (CC BY-SA 4.0); the ShareAlike clause enhances the open heritage and aims to create a larger open commons, ensuring that downstream users share back.

## We say we need standards, but do we use them?

The scientific community, funders and publishers all endorse the concept that common data and metadata standards underpin data reproducibility, ensuring that the relevant elements of a dataset are reported and shared consistently and meaningfully. However, navigating through the many standards available can be discouraging and often unappealing for prospective users. Bound within a particular discipline or domain, reporting standards are fragmented, with gaps and duplications, thereby limiting their combined used. Although standards should stand alone, they should also function well together, especially to better support not only multidimensional data but also the aggregation of pre-existing datasets from one or more disciplines or domains. Understanding how they work or how to comply with them takes time and effort. Measuring the uptake of standards, however, is not trivial, and achieving a full picture is practically impossible.

FAIRsharing provides a snapshot of the standards landscape, which is dynamic and will continue to evolve as we engage with more communities and verify the information we house, add new resources, track their life-cycle status and usage in databases and repositories, and link out to examples of training material. FAIRsharing also plays a fundamental role in the activation of the decision-making chain, which is an essential step toward fostering the wider adoption of standards. When a standard is mature and appropriate standard-compliant systems become available, such as databases and repositories, these must then be channeled to the relevant stakeholder community, who in turn must recommend them (for example, in data policies)—and ultimately may require them—or use them (for example, to define a data management plan) to facilitate a high-quality research cycle.

As of February 2019, 166 of FAIRsharing’s 1,293 community standards are generic and multidisciplinary and the rest are discipline specific (encompassing life, agricultural, health, biomedical, environmental, humanities and engineering sciences).133 reporting guidelines (out of 154), 641 terminology artifacts (out of 728), 357 models/formats (out of 387), and 10 identifier schemata (out of 11) are mature and tagged as ‘Ready’ for use. Table 1 displays the top ten most-accessed data and metadata standard records in FAIRsharing during 2018. This ranking most likely reflects the popularity of a standard rather than directly correlating with the level of standard adoption (by journal and funder data policies, or by databases and repositories). The ranking is also very variable and can change substantially from year to year, which may reflect the differing levels of activity focused on standard development in a particular research community over time.

**Table 1:** As of February 2019, the 12 data and metadata standards in the top ten positions (all tagged as ‘Ready’) ranked according to the page views in 2018 and subsequently ordered by the number of journals or publishers recommending them

Rank	Name	Type	Page views in 2018	Number of journals’ and publishers’ policies recommending it	Number of databases and repositories implementing it
1	Clinical Data Interchange Standards Consortium (CDISC) Analysis Data Model (ADaM) 10.25504/FAIRsharing.dvxkzb	Model/format	287	0	0
2	Minimum Information about any (x) Sequence (MIxS) 10.25504/FAIRsharing.9aa0zp	Reporting guideline	284	3	8
3	Minimum Information About a Microarray Experiment (MIAME) 10.25504/FAIRsharing.32b10v	Reporting guideline	247	2	11
4	Minimum Information about a high-throughput nucleotide SEQuencing Experiment (MINSEQE) 10.25504/FAIRsharing.a55z32	Reporting guideline	246	1	4
5	The FAIR Principles (FAIR) https://fairsharing.org/FAIRsharing.WWI10U	Reporting guideline	214	0^a^	2^a^
6	Minimum Information about a Flow Cytometry Experiment (MIFlowCyt) 10.25504/FAIRsharing.kcnjj2	Reporting guideline	170	0	2
7	Schema.org https://fairsharing.org/FAIRsharing.hzdzq8	Model/format	163	0	29
8	Gene Ontology (GO) 10.25504/FAIRsharing.6xq0ee	Terminology artifact	149	0	159
9	Core Attributes of Biological Databases (BioDBCore) 10.25504/FAIRsharing.qhn29e	Reporting guideline		0	2
10	DataCite Metadata Schema 10.25504/FAIRsharing.me4qwe	Model/format	145	7	14

^a^Although almost universally accepted, the use of the FAIR principles is implicit. FAIRsharing is working with both policy makers and repositories to raise awareness of the FAIR principles and we therefore expect these numbers to rise in the coming years.

Table 2 displays the top ten data and metadata standard records that have been implemented by databases and repositories, providing a realistic measure of the use of data and metadata standards to annotate, structure and share datasets. Surprisingly, with the exception of one (the US National Center for Biotechnology Information (NCBI) Taxonomy, a terminology artifact for taxonomic information: 10.25504/FAIRsharing.fj07xj), none of the other nine standards is explicitly recommended in journals and databases’ data policies, including the standard most implemented by databases and repositories (the FASTA Sequence Format, a model/format for representing either nucleotide sequences or peptide sequences: 10.25504/FAIRsharing.rz4vfg). This omission can probably be explained by the fact that, created in 1985, this is a de facto standard that every sequence database and repository implements by default, thus becoming (positively) ‘invisible’ to users, including publishers and journals.

**Table 2:** As of February 2019, the top ten data and metadata standards (all tagged ‘Ready’) ranked according to the number of implementations by databases and repositories

Rank	Name	Type	Number of databases and repositories implementing it	Number of journals’ and publishers’ policies recommending it	Page views in 2018
1	FASTA Sequence Format 10.25504/FAIRsharing.rz4vfg	Model/format	253	0	149
2	Gene Ontology (GO) 10.25504/FAIRsharing.6xq0ee	Terminology artifact	159	0	149
3	Protein Data Bank (PDB) Format 10.25504/FAIRsharing.9y4cqw	Model/format	59	0	10
4	Generic Feature Format Version 3 (GFF3) 10.25504/FAIRsharing.dnk0f6	Model/format	48	0	7
5	Chemical Entities of Biological Interest (ChEBI) 10.25504/FAIRsharing.62qk8w	Terminology artifact	35	0	35
6	NCBI Taxonomy (NCBITAXON) 10.25504/FAIRsharing.fj07xj	Terminology artifact	32	3	104
7	GenBank Sequence Format 10.25504/FAIRsharing.rg2vmt	Model/format	29	0	39
8	Schema.org 10.25504/FAIRsharing.hzdzq8	Model/format	29	0	119
9	Sequence Ontology (SO) 10.25504/FAIRsharing.6bc7h9	Terminology artifact	28	0	15
10	Molecular Interaction Tabular (MITAB) 10.25504/FAIRsharing.ve0710	Model/format	18	0	13

To understand how journals and publishers select which resource to recommend (https://fairsharing.org/recommendations), we have worked closely with the editors from the following eight journals or publishers: EMBO Press, *F1000Research*, Oxford University Press’s *GigaScience*, PLOS, Elsevier and Springer Nature’s BioMed Central and *Scientific Data*. As shown in Table 3 (https://fairsharing.org/article/live_list_standards_in_policies), as of February 2019, the 13 data policies of these journals or publishers recommend a total of 33 standards: 18 reporting guidelines, 8 terminology artifacts and 7 models/formats. Surprisingly, out of these 33, only 1 (the NCBI Taxonomy) is in the top ten standards most implemented by databases and repositories (as shown in Table 1), whereas one-third (10 reporting guidelines and 1 terminology artifact) are not even implemented. Furthermore, these data policies recommend 187 (generalist and domain-specific) databases and repositories. The 26 that occupy the top five positions are shown in Table 4 (https://fairsharing.org/article/live_list_databases_in_policies). As expected, this top tier includes public databases and repositories from major research and infrastructure providers from the United States and Europe; the domain-specific UniProt Knowledgebase (10.25504/FAIRsharing.s1ne3g) is at the top of the list with the higher number of standards implemented. However, this analysis also indicates that an additional 185 standards that are implemented by the recommended databases and repositories are not explicitly mentioned at all in these 13 journals’ or publishers’ data policies.

**Table 3:** As of February 2019, the 33 reporting guidelines in the top five positions (all tagged ‘Ready’) ranked according to the number of recommendations by 13 journals’ or publishers’ data policies (see main text) and subsequently ordered by the number of databases and repositories that implement them

Rank	Name	Type	Number of journals’ and publishers’ policies recommending it	Number of databases and repositories implementing it	Page views in 2018
1	FORCE11 Data Citation Principles (FORCE11 DC) 10.25504/FAIRsharing.9hynwc	Reporting guideline	9	3	27
	Animals in Research: Reporting In Vivo Experiments (ARRIVE) 10.25504/FAIRsharing.t58zhj	Reporting guideline	9	0	60
	CONSOlidated standards of Reporting Trials (CONSORT) 10.25504/FAIRsharing.gr06tm	Reporting guideline	9	0	36
	Preferred Reporting Items for Systematic reviews and Meta-Analyses (PRISMA) 10.25504/FAIRsharing.gp3r4n	Reporting guideline	9	0	39
	Case Reports (CARE) 10.25504/FAIRsharing.zgqy0v	Reporting guideline	9	0	17
2	DataCite Metadata Schema 10.25504/FAIRsharing.me4qwe	Model/format	7	14	85
3	NCBI Taxonomy (NCBITAXON) 10.25504/FAIRsharing.fj07xj	Terminology artifact	3	32	104
	Investigation Study Assay Tabular (ISA-Tab) 10.25504/FAIRsharing.53gp75	Model/format	3	11	67
	Minimum Information about any (x) Sequence (MIxS) 10.25504/FAIRsharing.9aa0zp	Reporting guideline	3	8	239
4	Minimum Information About a Microarray Experiment (MIAME) 10.25504/FAIRsharing.32b10v	Reporting guideline	2	11	162
	Minimum Information About a Proteomics Experiment (MIAPE) 10.25504/FAIRsharing.8vv5fc	Reporting guideline	2	4	62
	Minimum Information about a Molecular Interaction Experiment (MIMIx) 10.25504/FAIRsharing.8z3xzh	Reporting guideline	2	4	46
	MIAME Notation in Markup Language (MINiML) 10.25504/FAIRsharing.gaegy8	Model/format	2	2	32
	Consolidated criteria for reporting qualitative research (COREQ) 10.25504/FAIRsharing.6mhzhj	Reporting guideline	2	0	11
	STrengthening the Reporting of OBservational studies in Epidemiology (STROBE) 10.25504/FAIRsharing.1mk4v9	Reporting guideline	2	0	22
	STAndards for the Reporting of Diagnostic accuracy (STARD) 10.25504/FAIRsharing.956df7	Reporting guideline	2	0	15
	Consolidated Health Economic Evaluation Reporting Standards (CHEERS) 10.25504/FAIRsharing.neny94	Reporting guideline	2	0	10
	CONSOlidated Standards of Reporting Trials – Official Extensions (CONSORT-OE) 10.25504/FAIRsharing.wstthd	Reporting guideline	2	0	6
	CONsolidated Standards of Reporting Trials – Unofficial Extensions (CONSORT-UE) 10.25504/FAIRsharing.2kq1fs	Reporting guideline	2	0	5
5	Systems Biology Markup Language (SBML) 10.25504/FAIRsharing.9qv71f	Model/format	1	15	51
	Ontology for Biomedical Investigations (OBI) 10.25504/FAIRsharing.284e1z	Terminology artifact	1	11	71
	PSI Molecular Interaction Controlled Vocabulary (PSI-MI CV) 10.25504/FAIRsharing.8qzmtr	Terminology artifact	1	9	7
	Experimental Factor Ontology (EFO) 10.25504/FAIRsharing.1gr4tz	Terminology artifact	1	8	21
	mz Markup Language (mzML) 10.25504/FAIRsharing.26dmba	Model/format	1	7	30
	Minimal Information Required In the Annotation of Models (MIRIAM) 10.25504/FAIRsharing.ap169a	Reporting guideline	1	5	18
	Environment Ontology (EnvO) 10.25504/FAIRsharing.azqskx	Terminology artifact	1	5	23
	Minimal Information about a high throughput SEQuencing Experiment (MINSEQE) 10.25504/FAIRsharing.a55z32	Reporting guideline	1	4	129
	CellML 10.25504/FAIRsharing.50n9hc	Model/format	1	3	25
	BioAssay Ontology (BAO) 10.25504/FAIRsharing.mye76w	Terminology artifact	1	1	6
	eagle-i Research Resource Ontology (ERO) 10.25504/FAIRsharing.nwgynk	Terminology artifact	1	1	5
	ThermoML 10.25504/FAIRsharing.7b0fc3	Model/format	1	1	12
	Units Ontology (UO) 10.25504/FAIRsharing.mjnypw	Terminology artifact	1	1	16

**Table 4:** As of February 2019, the 26 databases and repositories in the top five positions (all tagged ‘Ready’) ranked according to the number of recommendations by 13 journals’ or publishers’ data policies (see main text) and subsequently ordered by the number of standards implemented

Rank	Name	Number of journals’ and publishers’ policies recommending it	Number of standards implemented	Page views in 2018
1	UniProt Knowledgebase (UniProtKB) 10.25504/FAIRsharing.s1ne3g	13	16	116
	European Nucleotide Archive (ENA) 10.25504/FAIRsharing.dj8nt8	13	9	165
	ArrayExpress 10.25504/FAIRsharing.6k0kwd	13	7	173
	GenBank 10.25504/FAIRsharing.9kahy4	13	9	386
	FAIRsharing 10.25504/FAIRsharing.2abjs5	13	6	122
	Gene Expression Omnibus (GEO) 10.25504/FAIRsharing.5hc8vt	13	4	106
2	PRoteomics IDEntifications database (PRIDE) 10.25504/FAIRsharing.e1byny	12	14	73
	MetaboLights (MTBLS) 10.25504/FAIRsharing.kkdpxe	12	8	197
	PANGAEA – Data Publisher for Earth and Environmental Science 10.25504/FAIRsharing.6yw6cp	12	7	22
3	MGnify – EBI Metagenomics 10.25504/FAIRsharing.dxj07r	11	5	70
	Sequence Read Archive (SRA) 10.25504/FAIRsharing.g7t2hv	11	4	135
	figshare 10.25504/FAIRsharing.drtwnh	11	2	415
	Open Science Framework (OSF) 10.25504/FAIRsharing.g4z879	11	0	489
	OpenNeuro 10.25504/FAIRsharing.s1r9bw	11	1	85
	Database of Genomic Variants Archive (DGVA) 10.25504/FAIRsharing.txkh36	11	0	47
	European Variation Archive (EVA) 10.25504/FAIRsharing.6824pv	11	2	29
	Coherent X-ray Imaging Data Bank (CXIDB) 10.25504/FAIRsharing.y6w78m	11	2	30
4	The European Genome-phenome Archive (EGA) 10.25504/FAIRsharing.mya1ff	10	6	68
	The Cancer Imaging Archive (TCIA) 10.25504/FAIRsharing.jrfd8y	10	1	102
5	NCBI BioSample 10.25504/FAIRsharing.qr6pqk	9	3	12
	RCSB Protein Data Bank (RCSB PDB) 10.25504/FAIRsharing.2t35ja	9	2	29
	Crystallography Open Database (COD) 10.25504/FAIRsharing.7mm5g5	9	0	63
	NeuroVault 10.25504/FAIRsharing.rm14bx	9	1	9
	National Addiction & HIV Data Archive Program (NAHDAP) 10.25504/FAIRsharing.k34tv5	9	0	116
	NCBI Trace Archives 10.25504/FAIRsharing.abwvhp	9	0	31
	HUGO Gene Nomenclature Committee (HGNC) 10.25504/FAIRsharing.29we0s	9	0	10

If one looks instead at all 82 journals’ or publishers’ data policies curated in FAIRsharing (instead of just 13), one sees the same discrepancy. As of February 2019, only 66 data policies mention one or more specific standards (https://fairsharing.org/article/live_list_journal_policies); the minimal reporting guidelines are recommended 17 times as often as terminology artifacts and 12 times as often as models/formats (and model formats are heavily implemented by data repositories); databases are recommended 702 times, with 187 databases recommended in total, 44 times as often as models/formats.

Based on ongoing activity with the eight journals and publishers mentioned above, along with other interested parties such as *eLife*, Taylor & Francis Group, Wiley and Hindawi (https://fairsharing.org/communities), we understand this discrepancy in recommendation to be the consequence of a cautious approach to choosing which standard to recommend where thousands of (often competing) standards are available. It is understandable if journals or publishers do not overreach. Recommendation of a standard is often driven by the editor’s familiarity with one or more standards, notably for journals or publishers focusing on specific disciplines and areas of study, or the engagement with learned societies and researchers actively supporting and using certain standards. As a rule, beyond individuals involved in standards developments, the rest of a research community that journals or publishers serve is often not familiar with standards; indeed, many researchers often perceive standards as a hindrance to data reporting rather than a help. Therefore, the current trend is for journals or publishers to recommend generalist repositories and a core set of discipline-specific repositories, even though a bigger number of (public and global, project-driven, and institution-based) databases and repositories exist. Similarly, journals and publishers tend to recommend very few standards, and those they do are usually data citation standards or minimum reporting guidelines (the metadata standards more relevant to publication). The general opinion of these editors is that terminology artifacts and models/formats instead should emerge from a close collaboration between their developing community and the implementing repositories, and they should remain only implicitly suggested.

FAIRsharing, therefore, is positioned to highlight to journals or publishers, as well as researchers and other stakeholders, which terminology artifacts and models/formats, along with other standards, each database and repository implements. This, along with community indicators of use and maturity, as well as emerging global certifications, is essential to inform the selection or recommendation of relevant databases and repositories. FAIRsharing aims to increase the visibility, citation and credit of these community-driven standards, databases and repository efforts.

## The best standards are invisible and transparent

Standards for reporting of data and metadata are essential for data reuse, which drives scientific discovery and reproducibility. Minimal reporting guidelines are intended for human consumption and are usually narrative in form and therefore prone to ambiguities, making compliance and validation difficult and approximate. Many of these guidelines, however, already come with (or lead to the development of) associated models/formats and terminology artifacts, which are created to be machine readable (rather than for human consumption). These two types of standards ensure the datasets are harmonized in regard to structure, formatting and annotation, setting the foundation for the development of tools and repositories that enable transparent interpretation, verification, exchange, integrative analysis and comparison of (heterogeneous) data. The goal is to ensure the implementation of these standards in data annotation tools and data repositories, making these standards invisible to the end users.

Models/formats and terminology artifacts are essential to the implementation of the FAIR principles that emphasize enhancing the ability of machines to automatically discover and use data and metadata. In particular, the ‘computability’ of standards is core to the development of FAIR metrics to measure the level of compliance of a given dataset against the relevant metadata descriptors. These machine-readable standards provide the necessary quantitative and verifiable measures of the degree to which data meet these reporting guidelines. The latter, on their own, would just be statements of unverifiable good intentions of compliance to given standards.

Delivering tools and practices to create standards-based templates for describing datasets smarter and faster is essential, if we are to use these standards in the authoring of metadata for the variety of data types in the life sciences and other disciplines. FAIRsharing is already involved in ongoing community discussions around the need for common frameworks for disciplinary research data management protocols^8^. Furthermore, research activities to deliver machine-readable standards are already underway by the FAIRsharing team and collaborators^9^; all outputs will be freely shared for others to develop tools that would make it easy to check the compliance of data to standards.

## Committed to community service

The FAIRsharing mission is to increase guidance to consumers of standards, databases, repositories, and data policies, to accelerate the discovery, selection and use of these resources; and increase producer satisfaction in terms of resource visibility, reuse, adoption and citation. Box 1 illustrates community-provided exemplar use cases that drive our work. This is a major undertaking, but it is a journey we are not making alone.

Collaborative work is happening on many fronts. We are categorizing the records according to discipline and domain via two open application ontologies. This should facilitate more accurate browsing, discovery and selection. To improve our policy registry, we are disambiguating policies from individual journals and those from publishers that encompass groups of journals. This will increase the number of journals covered and more accurately represent the different data policy models being pursued by publishers. Selection and decision-making are being improved by the enrichment of indicators based on community-endorsed and discipline-specific criteria, such as FAIR metrics and FAIRness level. To maximize the ‘look-up service’ functionality and to connect the content to other registries and tools, we are creating customizable interfaces for human as well as programmatic access to the data. We are also expanding the existing network graph and creating new visually accessible statistics (https://fairsharing.org/summary-statistics). Finally, on a monthly basis, we are highlighting featured exemplar resources, as well as adding to the informational and educational material available on FAIRsharing.

Box 1 How FAIRsharing can help different stakeholders FAIRsharing offers benefits to several different stakeholders in the research endeavor. For example:

- Casey (**a researcher**) searches FAIRsharing to identify an established repository, recognized by the journal she plans to submit to, with restricted data access to deposit her sensitive datasets, as recommended by her funder’s data policy.
- Andrea (**a biocurator**) searches FAIRsharing for suitable standards to describe a set of experiments. He filters the results by disciplines, focusing on standards implemented by one or more data repositories, with available annotations tools. He also looks for examples of the most up-to-date version of the standards and the details of a person or support group to contact.
- Alex (**a standards developer**) creates and maintains a personalized collection page on FAIRsharing to list and showcase the set of standards developed by the grassroots standard organization she is the representative of. Alex registers the standards and/or claims existing records added by the FAIRsharing team, vetting the descriptions and/or enhancing them by adding indicators of maturity for the standards and indicating the repositories and tools implementing them. Alex’s grassroots organization uses the collection to maximize the visibility of their standards, promoting adoption outside their immediate community, also favoring reuse in and extensions to other areas.
- Sam (**a repository manager**) registers the data resource at FAIRsharing manually or programmatically, describing terms of deposition and access, adding information on the resource’s relationship to other repositories and use of standards, and assessing the level FAIRness of his data repository. He links the record to funding source(s) supporting the resources and the institute(s) hosting it, as well as his ORCID profile to get credit for his role as maintainer of a resource. Sam receives alerts if a publisher recommends the repository in a data policy, and uses the DOI assigned to the repository record to cite the evidence of adoption.
- Marion (**a policymaker**) registers a journal’s data policy in FAIRsharing, creating and maintaining an interrelated list of the repositories and standards recommended to the authors, to deposit and annotated data and other digital assets. Marion keeps the data policy up to date using visualization and comparison functionalities, and consulting the knowledge graph that offers an interactive view of the repositories, tools and standards, as well as receiving customized alerts (for example, when a repository has changed its data access terms or when a standard has been superseded by another).
- Lesley (**a data manager**) consults FAIRsharing when creating a data management plan to identify the most appropriate reporting guidelines, formats and terminologies for data types, and formally cites these community standards using their DOIs and/or the ‘how to cite this record’ statements provided for each resource.
- Robin (**a librarian**) supports research data use in FAIRsharing by enriching educational and training material to support scholars in the use of data standards, in their ability to conform to journal and funder policies, and in developing and providing guidance that increases researchers’ capability and skills, empowering them to organize and make their data FAIR.

## Guidance to stakeholders

**Fig. 1:**
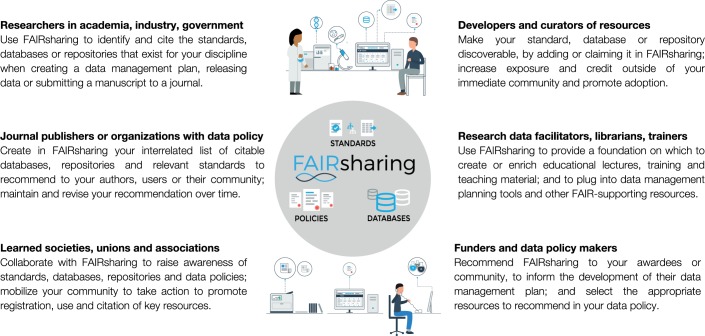
FAIRsharing guidance to each stakeholder group. Image by FAIRsharing.org, used under a Creative Commons BY-SA 4.0 license.

To foster a culture change within the research community into one where the use of standards, databases and repositories for FAIRer data is pervasive and seamless, we need to better promote the existence and value of these resources. First and foremost, we need to paint an accurate picture of the status quo. Several stakeholders can play catalytic roles (Fig. 1).

Standards developers and database curators can use FAIRsharing to explore what resources exist in their areas of interest (and whether those resources can be used or extended), as well as enhance the discoverability and exposure of their resource. This resource might then receive credit outside of their immediate community and ultimately promote adoption. (To learn how to add your resource to FAIRsharing or to claim it, see https://fairsharing.org/new.) A representative of a community standardization initiative is best placed to describe the status of a standard and to track its evolution. This can be done by creating an individual record (for example, the Data Documentation Initiative (DDI) standard for social, behavioral, economic, and health data; 10.25504/FAIRsharing.1t5ws6) or by grouping several records together in a collection (for example, the Human Proteome Organisation (HUPO) Proteomics Standards Initiative (PSI) standards for proteomics and interactomics data; https://fairsharing.org/collection/HUPOPSI). To achieve FAIR data, linked data models need to be provided that allow the publishing and connecting of structured data on the web. Similarly, representatives of a database or repository are uniquely placed to describe their resource and to declare the standards implemented (for example, the Inter-university Consortium for Political and Social Research (ICPSR) archive, which uses the DDI standard (10.25504/FAIRsharing.y0df7m); or the Reactome Knowledge Base (10.25504/FAIRsharing.tf6kj8), which uses several standards in the COmputational Modeling in BIology NEtwork (COMBINE) collection, https://fairsharing.org/collection/ComputationalModelingCOMBINE). The more adopted a resource is, the greater its visibility. For example, if your standard is implemented by a repository, these two records will be interlinked; thus, if someone is interested in that repository they will see that your standard is used by that resource. If your resource is recommended in a data policy from a journal, funder or other organization, it will be given a ‘recommended’ ribbon, which is present on the record itself and clearly visible when the resource appears in search results.

For journal publishers or organizations with a data policy, FAIRsharing enables the maintenance of an interrelated list of citable standards and databases, grouping those that the policy recommends to users or their community (for example, see examples of recommendations created by eight main publishers and journals; https://fairsharing.org/recommendations). As FAIRsharing continues to map the landscape, journals and publishers can also revise their selections over time, enabling the recommendation of additional resources with more confidence. All journals that do not have such data statements should develop them to ensure all data relating to an article or project are as FAIR as possible. Finally, journal editors should also encourage authors to cite the standards, database and repositories they use or develop via the ‘how to cite this record’ statement, found on each FAIRsharing record, which includes a DOI.

Trainers, educators, librarians and those organizations and services involved in supporting research data can use FAIRsharing to provide a foundation on which to create or enrich educational lectures, training and teaching material, and to plug it into data management planning tools. These stakeholder communities play a pivotal role to prepare the new generation of scientists and deliver courses and tools that address the need to guide or empower researchers to organize data and to make it FAIR.

Learned societies, international scientific unions and associations, and alliances of these organizations should raise awareness around standards, databases, repositories and data policies—in particular, on their availability, scope and value for FAIR and reproducible research. FAIRsharing works with many organizations that have already mobilized their community members to take action (for example, see refs. ^10–12^), to promote the use and adoption of key resources, and to initiate new or participate in existing initiatives to define and implement policies and projects.

Funders can use FAIRsharing to help select the appropriate resources to recommend in their data policy and highlight those resources that awardees should consider when writing their data management plan (for example, see ref. ^13^). Funders should recognize standards, as well as databases and repositories, as digital objects in their own right, which have and must have their own associated research, development and educational activities^14^. FAIRsharing has already been identified as a key resource and service that helps in turning FAIR data a reality^15^. New funding frameworks need to be created to provide catalytic support for the technical and social activities around standards, in specific domains, within and across disciplines to enhance their implementation in databases and repositories, and the interoperability and reusability of data.

Last but not least, researchers can use FAIRsharing as a lookup resource to identify and cite the standards, databases or repositories that exist for their data and discipline—for example, when creating a data management plan for a grant proposal or funded project, or when submitting a manuscript to a journal, to identify the recommended databases and repositories, as well as the standards they implement to ensure all relevant information about the data is collected at the source. Today’s data-driven science, as well as the growing demand from governments, funders and publishers for FAIRer data, requires greater researcher responsibility. Acknowledging that the ecosystem of guidance and tools is still work in progress, it is essential that researchers develop or enhance their research data management skills, or seek the support of professionals in this area.

FAIRsharing brings the producers and consumers of standards, databases, repositories and data policies closer together, with a growing list of adopters (https://fairsharing.org/communities). Representatives of institutions, libraries, journal publishers, funders, infrastructure programs, societies and other organizations or projects (that in turn serve and guide individual researchers or other stakeholders on research data management matters) can become adopters.

We welcome collaborative proposals and are open to participate in joint projects to develop services for specific stakeholders and communities. Join us or reach out to us, and let’s pave the way for FAIRer data together.

## Supplementary Information

Supplementary Information Supplementary Note

## References

[1] McMurry JA (2017). PLoS Biol..

[2] Cousijn H (2018). Sci. Data.

[3] Taylor CF (2008). Nat. Biotechnol..

[4] Field D (2009). Science.

[5] McQuilton P (2016). Database (Oxford).

[6] Wilkinson MD (2016). Sci. Data.

[7] Wilkinson MD (2018). Sci. Data.

[8] Science Europe. Presenting a framework for discipline-specific research data management. Science Europe Guidance Document D/2018/13.324/1. https://www.scienceeurope.org/wp-content/uploads/2018/01/SE_Guidance_Document_RDMPs.pdf (2018).

[9] Musen MA (2015). J. Am. Med. Inform. Assoc..

[10] CODATA, Participants of the First ICSU-CODATA Workshop on Data Standards. Exploiting the digital revolution: developing capacity and integrating data across the disciplines of science. *Zenodo*10.5281/zenodo.1193642 (2018).

[11] Allen, R. & Hartland, D. FAIR in practice – Jisc report on the Findable Accessible Interoperable and Reusable Data Principles (Version 1). *Zenodo*10.5281/zenodo.1245568 (2018).

[12] Shi L (2017). Nat Biotechnol..

[13] European Research Council: Scientific Council. Open research data and data management plans information for ERC grantees (version 2.0 24). https://erc.europa.eu/sites/default/files/document/file/ERC_info_document-Open_Research_Data_and_Data_Management_Plans.pdf (2018).

[14] Sansone, S.-A. & Rocca-Serra, P. Interoperatibility standards: digital objects in their own right. *Figshare*10.6084/m9.figshare.4055496.v1 (2016).

[15] Directorate General for Research and Innovation (European Commission). Turning FAIR into reality. 10.2777/1524 (2018).

