# Supplementary material for: FAIRsharing as a community approach to standards, repositories and policies
Source: Nat Biotechnol. 2019 Apr 2;37(4):358–67. doi: 10.1038/s41587-019-0080-8 (PMC6785156; doi:10.1038/s41587-019-0080-8)
Supplement: Supplementary file 1 — Supplementary Note [file 41587_2019_80_MOESM1_ESM.pdf]

nature biotechnology

SUPPLEMENTARY INFORMATION

In the format provided by the authors and unedited.

FAIRsharing as a community approach to standards, repositories and policies

Editor's note: This paper has been peer-reviewed.

Susanna-Assunta Sansone[1*], Peter McQuilton[1], Philippe Rocca-Serra[1], Alejandra Gonzalez-Beltran[1], Massimiliano Izzo[1], Allyson L. Lister[1], Milo Thurston[1] and the FAIRsharing Community[2]

[1]Oxford e-Research Centre, Department of Engineering Science, University of Oxford, Oxford, UK. [2]A complete list of members and affiliations appears in the Supplementary Note.
*e-mail: susanna-assunta.sansone@oerc.ox.ac.uk

Supplementary information to: FAIRsharing, a cohesive community approach to the growth in standards, repositories and policies.

Susanna-Assunta Sansone (0000-0001-5306-5690) Oxford e-Research Centre, Department of Engineering Science, University of Oxford, Oxford, OX1 3QG, UK.
susanna-assunta.sansone@oerc.ox.ac.uk
Peter McQuilton (0000-0003-2687-1982) Oxford e-Research Centre, Department of Engineering Science, University of Oxford, Oxford, OX1 3QG, UK.
Philippe Rocca-Serra (0000-0001-9853-5668) Oxford e-Research Centre, Department of Engineering Science, University of Oxford, Oxford, OX1 3QG, UK.
Alejandra Gonzalez-Beltran (0000-0003-3499-8262) Oxford e-Research Centre, Department of Engineering Science, University of Oxford, Oxford, OX1 3QG, UK.
Massimiliano Izzo (0000-0002-8100-6142) Oxford e-Research Centre, Department of Engineering Science, University of Oxford, Oxford, OX1 3QG, UK.
Allyson L. Lister (0000-0002-7702-4495) Oxford e-Research Centre, Department of Engineering Science, University of Oxford, Oxford, OX1 3QG, UK.
Milo Thurston (0000-0002-6468-9260) Oxford e-Research Centre, Department of Engineering Science, University of Oxford, Oxford, OX1 3QG, UK.

The FAIRsharing community (https://fairsharing.org/communities):

Dominique Batista (0000-0002-2109-489X) Oxford e-Research Centre, Department of Engineering Science, University of Oxford, Oxford, OX1 3QG, UK.
Ramon Granell (0000-0001-9572-0972) Oxford e-Research Centre, Department of Engineering Science, University of Oxford, Oxford, OX1 3QG, UK.
Melanie Adekale (0000-0002-3791-7360) MD Health Consulting Ltd, 95 Bilton Road, Rugby, CV22 7AS, UK.
Delphine Dauga (0000-0003-3152-1194) Bioself Communication, 28 rue de la Bibliothèque, 13001 Marseille, France.
Emma Ganley (0000-0002-2557-6204) PLOS (Public Library of Science), Carlyle House, Carlyle Road, Cambridge CB4 3DN, UK.
Simon Hodson (0000-0003-3179-7270) Committee on Data of the International Council for Science (CODATA), 5 rue Auguste Vacquerie, 75016 Paris, France.
Rebecca Lawrence (0000-0003-4817-8206) F1000, Middlesex House, 34-42 Cleveland St, Fitzrovia, London W1T 4LB
Varsha K. Khodiyar (0000-0002-2743-6918) Springer Nature, 4 Crinan Street, London, N1 9XW, UK.
Jessica D. Tenenbaum (0000-0003-3532-565X) Duke University School of Medicine, Department of Biostatistics & Bioinformatics, DUMC Box 2721, Durham, NC 27710 USA.
Myles Axton (0000-0002-8042-4131) Wiley, 111 River St. Hoboken, NJ, 07030, USA.

Michael Ball (0000-0003-4139-0802) Economic and Social Research Council (ESRC), Polaris House, North Star Avenue, Swindon, SN2 1UJ, UK.
Sébastien Besson (0000-0001-8783-1429) Open Microscopy Environment (OME), School of Life Sciences, University of Dundee, Dundee, DD1 5EH, Scotland, UK.
Theodora Bloom (0000-0002-0222-4177) The BMJ, BMA House, Tavistock Square, London, WC1H 9JR, UK.
Vivien Bonazzi (0000-0002-7359-9072) Office of the Director, National Institutes of Health, 8600 Rockville Pike, Rm 2S-20, Bethesda, MD, 20894, USA.
Rafael C. Jimenez (0000-0001-5404-7670) ELIXIR Hub, Wellcome Genome Campus, Hinxton, Cambridge, CB10 1SD, UK.
David Carr (0000-0003-1435-307X) Wellcome Trust, 215 Euston Rd, London, NW1 2BE, UK.
Wei Mun Chan (0000-0002-9971-813X) eLife Sciences Publications, Ltd, Westbrook Centre, Milton Road, Cambridge CB4 1YG, UK.
Caty Chung (0000-0001-7539-0857) University of Miami, Center for Computational Science, 1320 S Dixie Hwy, Coral Gables, Florida 33146, USA.
Daniel Clarke (0000-0003-3471-7416) Department of Pharmacological Sciences, Mount Sinai Center for Bioinformatics, Icahn School of Medicine at Mount Sinai, One Gustave L. Levy Place, Box 1603, New York, NY 10029, USA.
Geraldine Clement-Stoneham (0000-0001-9305-7230) Medical Research Council, 14th Floor, One Kemble Street, London WC2B 4AN, UK.
Helena Cousijn (0000-0001-6660-6214) DataCite, Welfengarten 1b, 30167 Hannover, Germany.
Michel Dumontier (0000-0003-4727-9435) Institute of Data Science Maastricht University, Universiteitssingel 60, 6229 ER, Maastricht, The Netherlands.
Esther Dzalé Yeumo (0000-0001-5954-8415) INRA, UAR 1266 DIST Délégation Information Scientifique et Technique, Centre de recherche Ile-de-France-Versailles-Grignon, Versailles, 78000, France.
Scott Edmunds (0000-0001-6444-1436) GigaScience, BGI Hong Kong Ltd., 16 Dai Fu Street, Tai Po Industrial Estate, N.T., Hong Kong, China.
Nicholas Everitt (0000-0001-8343-8910) Taylor & Francis, Park Square, Milton Park, Abingdon, OX14 4RN, UK
Dominic Fripp (0000-0001-5352-4666) Jisc, One Castlepark, Tower Hill, Bristol, BS2 0JA, UK.
Carole Goble (0000-0003-1219-2137), School of Computer Science, The University of Manchester, Oxford Road, Manchester, M13 9PL, UK.
Martin Golebiewski (0000-0002-8683-7084) Convenor of ISO/TC 276/WG 5 Data Processing and Integration of the International Organization for Standardization (ISO), and Heidelberg Institute for Theoretical Studies (HITS), Schloss-Wolfsbrunnenweg 35, 69118 Heidelberg, Germany.
Neil Hall (0000-0003-2808-0009) ELIXIR UK, The Earlham Institute, Norwich Research Park, Norwich, NR4 7UZ, UK.
Robert Hanisch (0000-0002-6853-4602) Office of Data and Informatics, MS 6410, National

Institute of Standards and Technology (NIST), 100 Bureau Drive, Gaithersburg, MD 20899, USA.
Michael Hucka (0000-0001-9105-5960) Computing and Mathematical Sciences, California Institute of Technology, 1200 E. California Blvd, Pasadena, CA 91125, USA.
Mike Huerta (0000-0001-7787-8864) National Library of Medicine (NLM), National Institutes of Health (NIH), 8600 Rockville Pike, Rm 2S-20, Bethesda, MD, 20894, USA.
Amye Kenall (0000-0002-3030-8001) BMC, 4 Crinan Street, London, N1 9XW, UK.
Robert Kiley (0000-0003-4733-2558) Wellcome Trust, 215 Euston Rd, London. NW1 2BE, UK.
Juergen Klenk (0000-0002-7912-4801) Deloitte Consulting LLP, 1919 N Lynn St, Arlington, VA 22209, USA.
Dimitris Koureas (0000-0002-4842-6487) Biodiversity Information Standards Organisation (TDWG) & Naturalis Biodiversity Center, The Netherlands.
Jennie Larkin (0000-0003-0276-7822) National Institute of Diabetes, Digestive, and Kidney Diseases (NIDDK), National Institutes of Health (NIH), 6707 Democracy Blvd, Bethesda, MD 20817, USA.
Thomas Lemberger (0000-0002-2499-4025) EMBO Press, Meyerhofstrasse 1, 69117 Heidelberg, Germany.
Nick Lynch (0000-0002-8997-5298) Pistoia Alliance, 401 Edgewater Place, Suite 600. Wakefield MA 01880, USA.
Avi Ma'ayan (0000-0002-6904-1017) Department of Pharmacological Sciences, Mount Sinai Center for Bioinformatics, Icahn School of Medicine at Mount Sinai, One Gustave L. Levy Place, Box 1603, New York, NY 10029, USA.
Catriona McCallum (0000-0001-9623-2225) Hindawi Limited, 3rd Floor, Adam House, 1 Fitzroy Square, London W1T 5HF, UK.
Barend Mons (0000-0003-3934-0072) LUMC, President of CODATA, GO FAIR International Support & Coordination Office, Dutch office, Poortgebouw N-01, Rijnsburgerweg 10, 2333 AA Leiden, The Netherlands.
Josh Moore (0000-0003-4028-811X) Open Microscopy Environment (OME), School of Life Sciences, University of Dundee, Dundee, DD1 5EH, Scotland, UK.
Wolfgang Müller (0000-0002-4980-3512) Heidelberg Institute for Theoretical Studies (HITS), Schloss-Wolfsbrunnenweg 35, 69118 Heidelberg, Germany.
Holly Murray (0000-0002-8243-2493) F1000, Middlesex House, 34-42 Cleveland St, Fitzrovia, London W1T 4LB, UK.
Tomoe Nobusada (0000-0002-4133-1565) National Bioscience Database Center, Japan Science and Technology Agency, 5-3, Yonbancho, Chiyoda-ku, Tokyo 102-0081, Japan.
Daniel Noesgaard (0000-0002-0407-1805) GBIF | Global Biodiversity Information Facility - Secretariat, Universitetsparken 15, DK-2100 Copenhagen, Denmark.
Jennifer Paxton-Boyd (0000-0002-1296-5593) Oxford University Press, Great Clarendon Street, Oxford, OX2 6DP, UK.
Sandra Orchard (0000-0002-8878-3972) European Bioinformatics Institute (EMBL-EBI), European

Molecular Biology Laboratory, Wellcome Genome Campus, Hinxton, CB10 1SD, UK.
Gabriella Rustici (0000-0003-3085-1271) Department of Genetics, University of Cambridge, Downing Street, Cambridge CB2 3EH, UK.
Lynn M. Schriml (0000-0001-8910-9851) University of Maryland School of Medicine, Institute for Genome Sciences, Baltimore, Maryland 21201, USA.
Stephan Schürer (0000-0001-7180-0978) University of Miami, Department of Pharmacology, 1600 NW 10th Avenue, Miami, FL 33136, USA.
Kathryn Sharples (0000-0003-2809-6828) Wiley, 9600 Garsington Road, Oxford, OX4 2DQ, UK.
Marina Soares E Silva (0000-0001-9530-627X) Elsevier, Radarweg 29, 1043NX, Amsterdam, The Netherlands.
Imma Subirats (0000-0002-3401-8597) Food and Agriculture Organization of the United Nations (FAO), Viale delle terme di Caracalla, 00153 Rome, Italy.
Jason Swedlow (0000-0002-2198-1958) Open Microscopy Environment (OME), School of Life Sciences, University of Dundee, Dundee, DD1 5EH, Scotland, UK; Glencoe Software, Inc., 800 5th Ave, #101-259, Seattle, WA 98104, USA.
Marta Teperek (0000-0001-8520-5598) TU Delft Library, Prometheusplein 1, 2628 ZC Delft, Netherlands.
Weida Tong* (0000-0003-3488-6148) National Center for Toxicological Research, US Food and Drug Administration, 3900 NCTR Road, Jefferson, Arkansas, AR 72079, USA.
Mark Wilkinson (0000-0001-6960-357X) Center for Plant Biotechnology and Genomics UPM-INIA, Universidad Politécnica de Madrid, Pozuelo de Alarcón, 28223, Madrid, Spain.
John Wise (**0000-0002-5311-6555**) Pistoia Alliance, 401 Edgewater Place, Suite 600. Wakefield MA 01880, USA.
Pelin Yilmaz (0000-0003-4724-323X) Microbial Physiology Group, Max Planck Institute for Marine Microbiology, Celsiusstrasse 1, Bremen, Germany.

* Disclaimer: The views presented in this article do not necessarily reflect current or future opinion or policy of the US Food and Drug Administration. Any mention of commercial products is for clarification and not intended as an endorsement.
